# Impact of remnant healthy pulp and apical tissue on outcomes after simulated regenerative endodontic procedure in rat molars

**DOI:** 10.1038/s41598-020-78022-w

**Published:** 2020-12-01

**Authors:** Naoki Edanami, Kunihiko Yoshiba, Mari Shirakashi, Razi Saifullah Ibn Belal, Nagako Yoshiba, Naoto Ohkura, Aiko Tohma, Ryosuke Takeuchi, Takashi Okiji, Yuichiro Noiri

**Affiliations:** 1 Division of Cariology, Operative Dentistry and Endodontics, Department of Oral Health Science, Niigata University Graduate School of Medical and Dental Sciences, 2-5274 Gakkocho-dori, Chuo-ku, Niigata, 951-8514 Japan; 2 Division of Oral Science for Health Promotion, Department of Oral Health and Welfare, Niigata University Graduate School of Medical and Dental Sciences, Niigata, Japan; 3 Department of Pulp Biology and Endodontics, Division of Oral Health Sciences, Graduate School of Medical and Dental Sciences, Tokyo Medical and Dental University (TMDU), Tokyo, Japan

**Keywords:** Dental diseases, Immunohistochemistry

## Abstract

When regenerative endodontic procedures (REPs) are performed on immature teeth diagnosed with pulp necrosis and apical periodontitis, various healing patterns occur. Furthermore, infected immature teeth with endodontic disorders often exhibit some remnant pulp and apical tissue. Therefore, this study investigated the impact of remnant healthy or fully functional pulp and apical tissue on healing patterns after REPs. Simulated REPs were performed on non-infected immature rat molars with different amounts of remnant pulp and apical tissue. Healing patterns in these teeth were assessed after 28 days. Teeth with 0.81–0.91 mm of remnant pulp healed with pulp-like tissue, dentin, and osteodentin-like dentin-associated mineralized tissue (OSD-DAMT); teeth with 0.60–0.63 mm of remnant pulp healed with pulp-like tissue and OSD-DAMT; teeth with 0.13–0.43 mm of remnant pulp healed with periodontal ligament (PDL)-like tissue, OSD-DAMT, and cementum-like dentin-associated mineralized tissue (CEM-DAMT); and teeth with disorganization of pulp and apical tissues at 0.15–0.38 mm beyond the root apex healed with PDL-like tissue, CEM-DAMT, and intracanal bone (IB). Loss of Hertwig’s epithelial root sheath was observed with IB formation. These results showed that four distinct healing patterns occurred after REPs, depending on the preoperative amount of remnant healthy pulp and apical tissue.

## Introduction

Human teeth erupt into the oral cavity prior to complete root formation. Immature teeth can develop pulp necrosis and apical periodontitis due to dental caries and trauma. These endodontic disorders arrest further root formation and cause the teeth to become fragile and prone to fracture. Regenerative endodontic procedures (REPs) have attracted considerable attention as a new treatment approach for immature teeth diagnosed with pulp necrosis and apical periodontitis because they induce neo-tissue formation in the root canal and reinforce the tooth structure^1^.

Recently, histological and radiographic analyses of REPs have revealed non-uniform healing patterns after REPs on immature teeth diagnosed with pulp necrosis and apical periodontitis. A previous study demonstrated that newly formed intracanal connective tissue constitutes pulp-like tissue in REP-treated teeth^2^, whereas another study showed that this connective tissue constitutes periodontal ligament (PDL)-like tissue^3^. Furthermore, it has been shown that both dentin and cementum-like ectopic mineralized tissue (dentin-associated mineralized tissue: DAMT) or bone-like ectopic mineralized tissue (intracanal bone: IB) are formed in various combinations in REP-treated teeth^2,4,5^. Following REPs, regeneration of the dentin-pulp complex is ideal for the recovery of physiological functions such as dentin sensitivity and tooth immunity^6^. The formation of DAMT, with or without PDL-like intracanal connective tissue, is non-physiological; however, it is acceptable for REP-treated teeth because DAMT may strengthen the tooth structure^7,8^. In contrast, IB formation is undesirable for REP-treated teeth because of the risk of tooth-bone ankylosis and subsequent replacement root resorption^9^. Thus, the development of appropriate REP treatment protocols to ensure a desirable healing outcome is necessary for the management of immature teeth diagnosed with pulp necrosis and apical periodontitis. However, current protocols do not consistently result in predictable outcomes, because the mechanisms underlying the wide array of healing patterns have not yet been elucidated.

Previous clinical and histological studies have shown that immature teeth diagnosed with pulp necrosis and apical periodontitis do not necessarily lose all pulp and apical tissues; they often contain some remnant pulp and apical tissue^10–14^. These findings suggest that variations in healing patterns after REPs involving immature teeth diagnosed with pulp necrosis and apical periodontitis can be caused by differences in the preoperative amount of remnant pulp and apical tissue. Therefore, to begin elucidating the mechanism by which healing patterns are established after REPs on immature teeth diagnosed with pulp necrosis and apical periodontitis, the impacts of remnant healthy or fully functional pulp and apical tissue on healing patterns after REPs should be investigated.

Previous animal experiments involving REPs have been conducted in larger animals—including dogs^15^, ferrets^16^, and sheep^17^—because there are technical difficulties involved in performing REPs in smaller animals. However, REP experiments in rat molars may be possible by using a microscope and small instruments, based on a root canal treatment model in rat molars^18^. In rat molars, precise identification of mineralized and connective tissues can be performed by means of immunohistochemistry with well-characterized antibodies^19–22^. These methods enable detailed classification of healing patterns after REPs.

To the best of our knowledge, there have been no reports of methods to measure the amount of remnant pulp and apical tissue; however, this measurement might be achieved by visualizing tissue-deficient space in or around the root canal using micro-computed tomography (CT) scans with intracanal injection of X-ray contrast media.

Therefore, this study aimed to elucidate the relationship of healing patterns after REPs with the preoperative amount of remnant healthy pulp and apical tissue by performing simulated REPs on non-infected immature rat molars with different amounts of remnant healthy pulp and apical tissue, as well as by performing histological and immunohistochemical analyses of the healing patterns of these teeth.

## Results

### Baseline teeth and untreated control teeth

**Figure 1:**
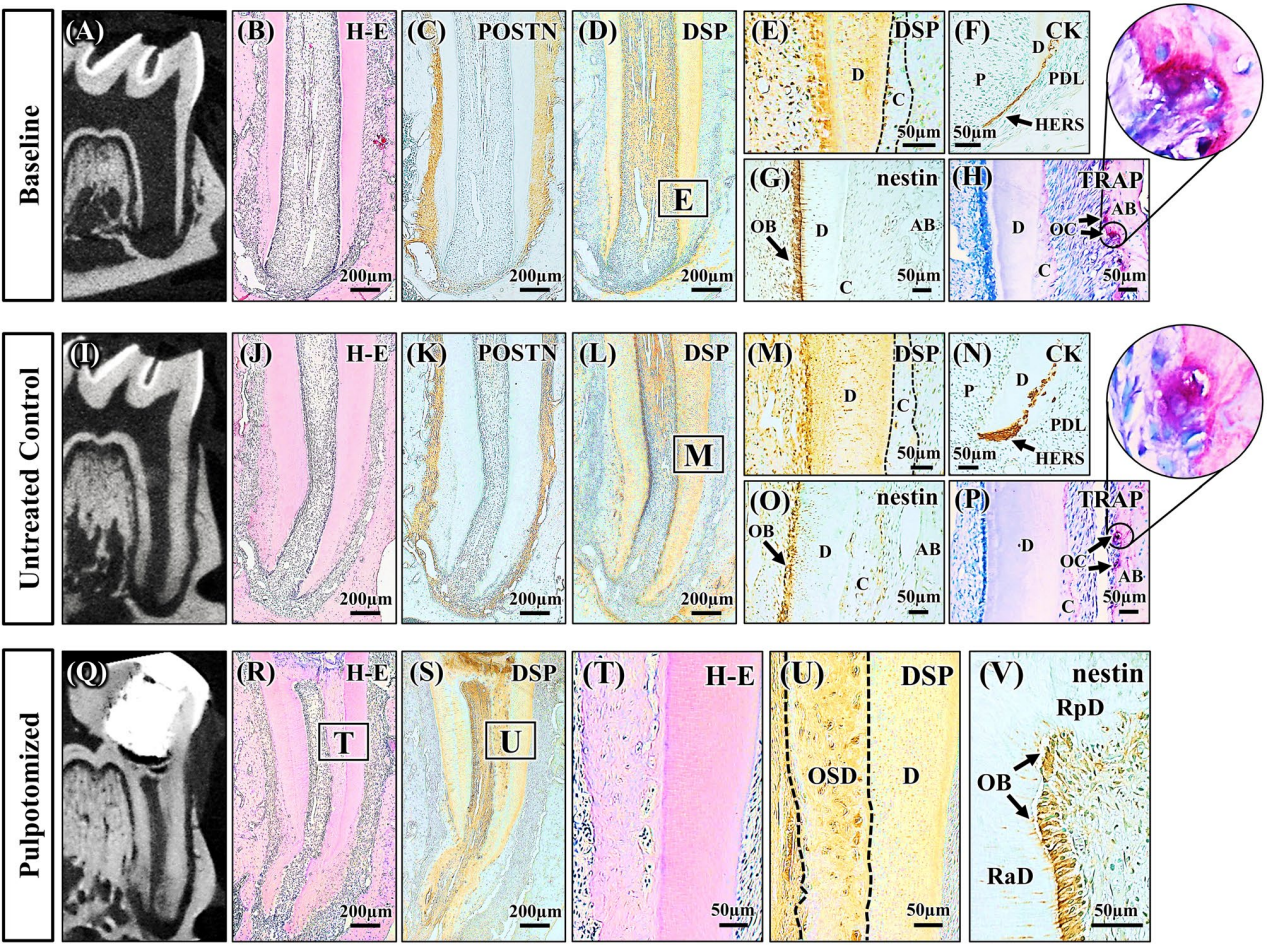
Baseline tooth, untreated control tooth, and pulpotomized tooth. (**A**,**B**,**I**,**J**) Micro-CT images and hematoxylin–eosin (H–E) staining images of baseline tooth and untreated control tooth. The baseline tooth exhibits immature roots with open apices and thin dentinal walls, while the untreated control tooth exhibits mature roots with closed apices and thicker dentinal walls. (**C**–**H**,**K**–**P**) Immunohistochemical staining images and tartrate-resistant acid phosphatase (TRAP) staining images of baseline tooth and untreated control tooth. Higher magnification views of boxed regions in D and L are shown in E and M, respectively. There are no differences in immunolocalization of analyzed proteins and localization of TRAP activity between the immature baseline tooth and the mature untreated control tooth. Periostin (POSTN) immunoreactivity is observed in periodontal ligament, but not in dental pulp (**C**,**K**). Dentin sialoprotein (DSP) immunoreactivity is observed in dentin, but not in cementum (with the exception of a few cementocytes) (**D**,**E**,**L**,**M**). Cytokeratin (CK) immunoreactivity is observed in bilayered Hertwig's epithelial root sheath at the root apex (**F**,**N**). Intense nestin immunoreactivity is limited to elongated odontoblasts (**G**,**O**). TRAP activity is limited to multinucleated osteoclasts on alveolar bone surface (**H**,**P**). (**Q**–**V**) Micro-CT images, H–E staining images, and immunohistochemical staining images of pulpotomized tooth. Higher magnification views of boxed regions in R and S are shown in T and U, respectively. Osteodentin is observed at the coronal third (**Q**,**R**,**T**); osteodentin is immunopositive for DSP (**S**,**U**). Nestin-positive odontoblasts are observed on the surfaces of reactionary and reparative dentin (**V**). *AB* Alveolar bone, *C* cementum, *D* dentin, *HERS* Hertwig's epithelial root sheath, *OB* odontoblast, *OC* osteoclast, *OSD* osteodentin, *P* pulp, *PDL* periodontal ligament, *RaD* reactionary dentin, *RpD* reparative dentin.

In this study, 5-week-old rats underwent simulated REPs on their left mandibular first molars and were sacrificed at 28 days after treatment. The baseline teeth (mandibular first molars of 5-week-old rats) and the untreated control teeth (right mandibular first molars of experimental rats) were radiographically, histologically, and immunohistochemically characterized. Baseline teeth exhibited immature roots (Fig. 1A,B), while untreated control teeth exhibited mature roots (Fig. 1I,J). No ectopic tissue was observed in the untreated control teeth (Fig. 1J). Analyzed proteins and tartrate-resistant acid phosphatase (TRAP) activity exhibited identical localization in immature baseline teeth and mature untreated control teeth. Periostin expression was confined to PDL tissue, while pulp tissue did not express periostin (Fig. 1C,K). Intense immunoreactivity for dentin sialoprotein (DSP) was observed in dentin; however, no immunoreactivity was detected in cementum, with the exception of a few cementocytes (Fig. 1D,E,L,M). Cytokeratin immunoreactivity was observed in bilayered Hertwig's epithelial root sheath (HERS) at root apices (Fig. 1F,N). Intense nestin immunoreactivity was limited to elongated odontoblasts, while minimal nestin immunoreactivity was observed in some cementoblasts and perivascular cells (Fig. 1G,O). TRAP activity was only observed in multinucleated osteoclasts on the alveolar bone surface (Fig. 1H,P). These localizations of analyzed proteins and TRAP activity were consistent with the results of previous studies regarding periostin^19^, DSP^20,23^, cytokeratin^21^, nestin^22^, and TRAP^24^.

### Pulpotomized teeth

To characterize mineralized tissues formed during the pulp wound healing process, pulpotomy was performed on mandibular first molars of 5-week-old rats. One pulpotomized tooth exhibited osteodentin, characterized by atubular structure and cells embedded within lacunae (Fig. 1Q,R,T). The osteodentin was immunopositive for DSP (Fig. 1S,U), consistent with the results of previous studies^25,26^. Reactionary and reparative dentin were immunopositive for DSP and lined with nestin-positive odontoblasts (Fig. 1S,V).

### REP-treated teeth

**Figure 2:**
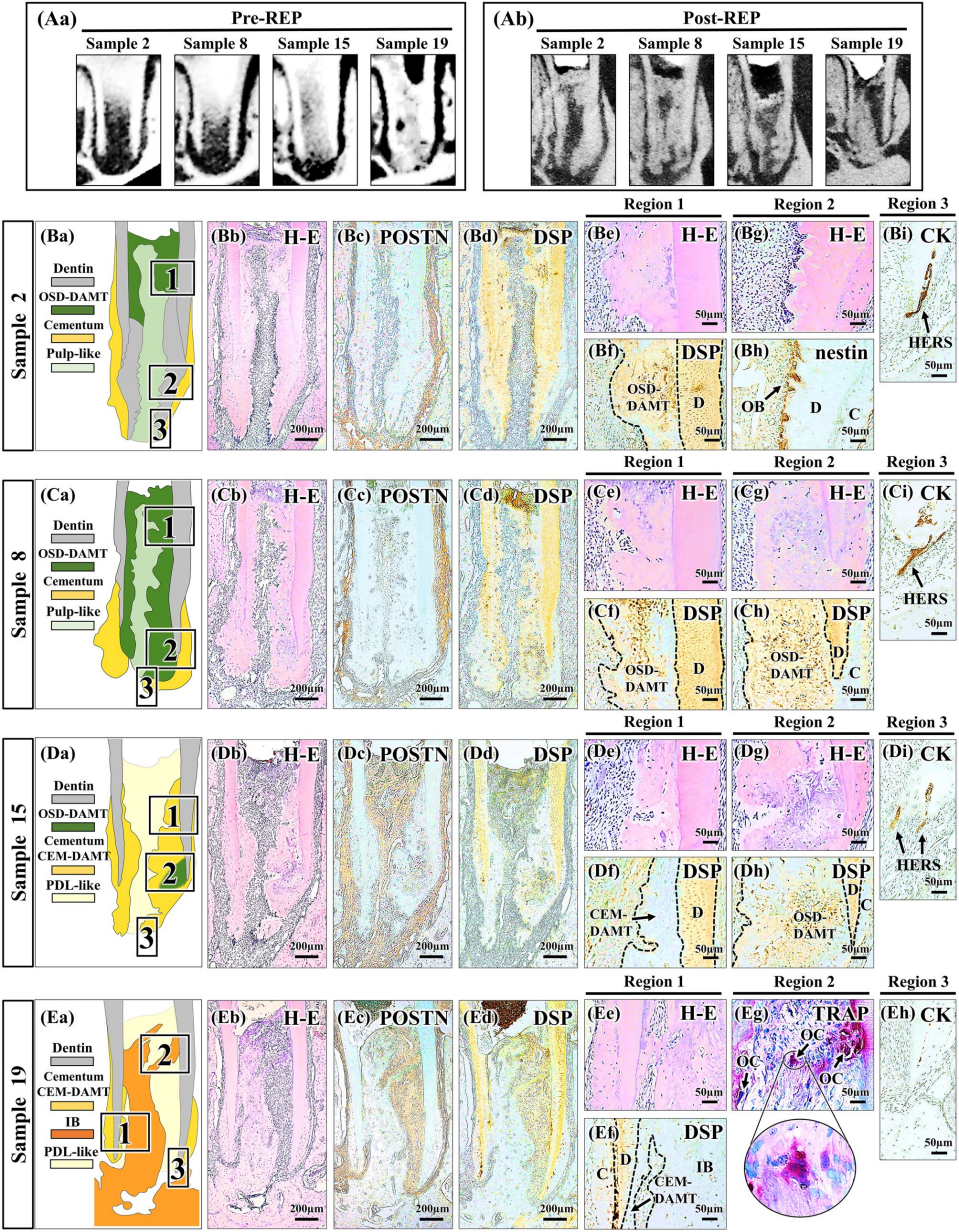
Four representative samples of regenerative endodontic procedure (REP)-treated teeth with distinct healing patterns. Sample numbers are indicated. (**Aa**,**Ab**) Pre- and post-REP micro-CT images. Mineralized tissue formation after REPs is apparent in the root canal. (**Ba**–**Bi**) Schematic diagram and histological/immunohistochemical images of sample 2. Imaging regions of **Be**–**Bi** are indicated in **Ba**. Newly formed mineralized tissue of the tooth comprises dentin and osteodentin-like dentin-associated mineralized tissue (OSD-DAMT) (**Bb**,**Bd**–**Bh**). Newly formed dentin exhibits tubular structures and nestin-positive odontoblasts on the surface (**Bg**,**Bh**). OSD-DAMT exhibits adherence to inner dentinal wall, embedded cells, and positive immunoreactivity for dentin sialoprotein (DSP) (**Be**,**Bf**). Intracanal tissue of the tooth comprises periostin (POSTN)-negative pulp-like tissue (**Bc**). Cytokeratin (CK)-positive Hertwig’s epithelial root sheath (HERS) is present at the root apex (**Bi**). (**Ca**–**Ci**) Schematic diagram and histological/immunohistochemical images of sample 8. Imaging regions of **Ce**–**Ci** are indicated in **Ca**. Newly formed mineralized tissue of the tooth comprises OSD-DAMT alone (**Cb**,**Cd**–**Ch**). Intracanal tissue of the tooth comprises POSTN-negative pulp-like tissue (**Cc**). CK-positive HERS is present at the root apex (**Ci**). (**Da**–**Di**) Schematic diagram and histological/immunohistochemical images of sample 15. Imaging regions of **De**–**Di** are indicated in **Da**. Newly formed mineralized tissue of the tooth comprises cementum-like dentin-associated mineralized tissue (CEM-DAMT) and OSD-DAMT (**Db**,**Dd**–**Dh**). CEM-DAMT exhibits adherence to inner dentinal wall, embedded cells, and negative immunoreactivity for DSP (**De**,**Df**). Intracanal tissue of the tooth comprises POSTN-positive periodontal ligament (PDL)-like tissue (**Dc**). CK-positive HERS is present at the root apex (**Di**). (**Ea**–**Eh**) Schematic diagram and histological/immunohistochemical images of sample 19. Imaging regions of **Ee**–**Eh** are indicated in **Ea**. Newly formed mineralized tissue of the tooth comprises CEM-DAMT and intracanal bone (IB) (**Eb**,**Ed**–**Eg**). IB exhibits continuity with apical alveolar bone and tartrate-resistant acid phosphatase (TRAP)-positive osteoclasts on the surface (**Eb**,**Eg**). Intracanal tissue of the tooth comprises POSTN-positive PDL-like tissue (**Ec**). CK-positive HERS is absent from the root apex (**Eh**). *C* Cementum, *D* dentin, *H–E* hematoxylin–eosin, *OB* odontoblast, *OC* osteoclast.

Comparing Pre- and post-REP micro-CT images, neo-tissue formation after REPs was apparent in the root canal (Fig. 2Aa,Ab). Mineralized and connective tissue components of REP-treated teeth were identified by reference to the histological results of immature baseline teeth, mature untreated control teeth, and pulpotomized teeth. There were four types of newly formed mineralized tissue. Mineralized tissue that exhibited tubular structures and nestin-positive odontoblasts on the surface was identified as newly formed dentin (Fig. 2Bg,Bh). No nestin-positive odontoblasts were observed on the surfaces of other newly formed mineralized tissues (Supplementary Fig. S1). Mineralized tissue that exhibited embedded cells and adherence to the inner dentinal wall was identified as DAMT, in accordance with the findings of previous studies^16,27^. The DAMT was further divided into DSP-positive and DSP-negative subtypes (Fig. 2Bb,Bd–Bf,Cb,Cd–Ch,Db,Dd–Dh,Eb,Ed–Ef). DSP-positive DAMT was designated as osteodentin-like DAMT (OSD-DAMT) and DSP-negative DAMT was designated as cementum-like DAMT (CEM-DAMT) because DAMT structurally resembles osteodentin or cementum; notably, osteodentin was immunopositive for DSP (Fig. 1S,U), while cementum was immunonegative for DSP (Fig. 1D,E,L,M). Mineralized tissue that exhibited continuity with alveolar bone and TRAP-positive osteoclasts on the surface was designated as IB (Fig. 2Eb,Eg), in accordance with the findings of previous studies^9,15,28^. No TRAP-positive osteoclasts were observed on the surfaces of other newly formed mineralized tissues (Supplementary Fig. S2). These four types of mineralized tissues formed in the following combinations: newly formed dentin with OSD-DAMT, OSD-DAMT alone, CEM-DAMT with OSD-DAMT, and CEM-DAMT with IB (Fig. 2Ba,Ca,Da,Ea), in 25%, 20%, 30%, and 25% of specimens, respectively. The type of mineralized tissue formation was clearly associated with the preoperative amount of remnant healthy pulp and periapical tissue, as noted below. Periostin immunostaining revealed that the intracanal tissue comprised periostin-negative pulp-like tissue in teeth with newly formed dentin and OSD-DAMT, as well as in teeth with OSD-DAMT alone (Fig. 2Bc,Cc); conversely, the intracanal tissue comprised periostin-positive PDL-like tissue in teeth with CEM-DAMT and OSD-DAMT, as well as in teeth with CEM-DAMT and IB (Fig. 2Dc,Ec). Cytokeratin immunostaining revealed that cytokeratin-positive HERS was absent only from teeth with CEM-DAMT and IB (Fig. 2Bi,Ci,Di,Eh). Thus, four distinct healing patterns were characterized.

### Relationship of healing patterns after REPs with preoperative amount of healthy pulp and apical tissue remnants

**Figure 3:**
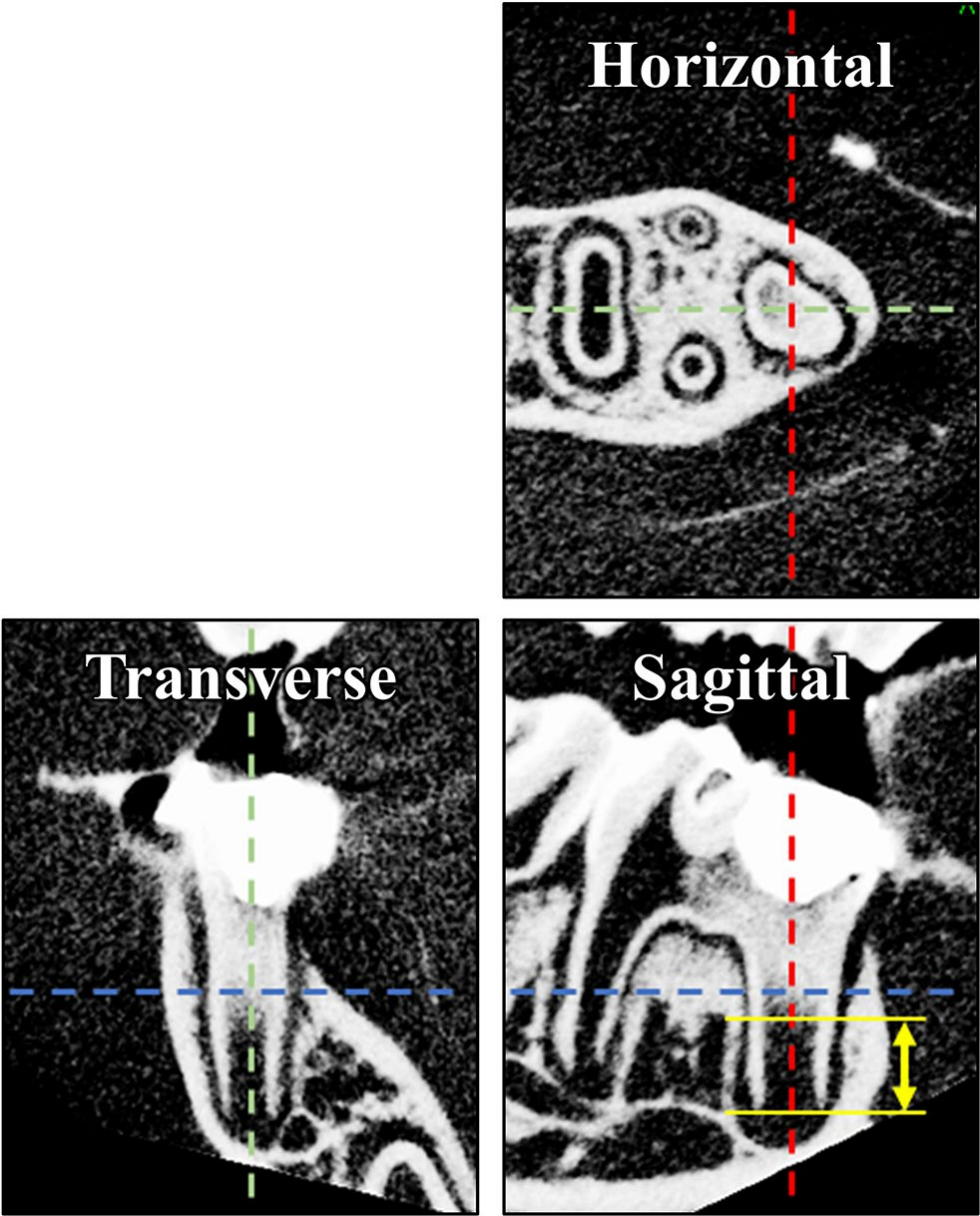
Standardization of micro-CT images and measurement of preoperative amount of remnant pulp and apical tissue. The x-axis (blue line) passes through the coronal third of the mesial root, the y-axis (red line) passes through the center of the mesial root, and the z-axis (green line) passes through the centers of the mesial and distal roots. Distance from the apical foramen to the bottom of the contrast-enhanced area (yellow double-sided arrow) is measured in sagittal images and recorded as the preoperative amount of remnant healthy pulp and apical tissue.

The amounts of healthy pulp and apical tissue remnants before simulated REPs were measured in pre-REP contrast enhanced micro-CT images (Fig. 3). Teeth with 0.81–0.91 mm of remnant healthy pulp healed with reconstruction of pulp-like tissue, as well as formation of dentin and OSD-DAMT; teeth with 0.60–0.63 mm of remnant healthy pulp healed with reconstruction of pulp-like tissue and formation of OSD-DAMT alone; teeth with 0.13–0.43 mm of remnant healthy pulp healed with replacement of intracanal tissue by PDL-like tissue and formation of both CEM-DAMT and OSD-DAMT; and teeth with disorganization of pulp and apical tissues at 0.15–0.38 mm beyond the root apex healed with replacement of intracanal tissue by PDL-like tissue, loss of HERS, and formation of both CEM-DAMT and IB (Table 1).

**Table 1:** Relationship of healing patterns after regenerative endodontic procedures with preoperative amount of remnant healthy pulp and apical tissue.

Sample number	Amount of remnant pulp and apical tissue	Healing pattern		
		Newly formed mineralized tissue	Intracanal tissue	Existence of HERS
1	0.91 mm	Dentin and OSD-DAMT	Pulp-like	Present
2	0.90 mm	Dentin and OSD-DAMT	Pulp-like	Present
3	0.86 mm	Dentin and OSD-DAMT	Pulp-like	Present
4	0.84 mm	Dentin and OSD-DAMT	Pulp-like	Present
5	0.81 mm	Dentin and OSD-DAMT	Pulp-like	Present
6	0.63 mm	OSD-DAMT	Pulp-like	Present
7	0.62 mm	OSD-DAMT	Pulp-like	Present
8	0.62 mm	OSD-DAMT	Pulp-like	Present
9	0.60 mm	OSD-DAMT	Pulp-like	Present
10	0.43 mm	CEM-DAMT and OSD-DAMT	PDL-like	Present
11	0.30 mm	CEM-DAMT and OSD-DAMT	PDL-like	Present
12	0.23 mm	CEM-DAMT and OSD-DAMT	PDL-like	Present
13	0.21 mm	CEM-DAMT and OSD-DAMT	PDL-like	Present
14	0.15 mm	CEM-DAMT and OSD-DAMT	PDL-like	Present
15	0.13 mm	CEM-DAMT and OSD-DAMT	PDL-like	Present
16	− 0.15 mm	CEM-DAMT and IB	PDL-like	Absent
17	− 0.28 mm	CEM-DAMT and IB	PDL-like	Absent
18	− 0.30 mm	CEM-DAMT and IB	PDL-like	Absent
19	− 0.30 mm	CEM-DAMT and IB	PDL-like	Absent
20	− 0.38 mm	CEM-DAMT and IB	PDL-like	Absent

Negative number indicates amount of tissue disorganization beyond root apex.

*OSD-DAMT* Osteodentin-like dentin-associated mineralized tissue, *CEM-DAMT* cementum-like dentin-associated mineralized tissue, *IB* intracanal bone, *PDL* periodontal ligament, *HERS* Hertwig's epithelial root sheath.

## Discussion

REPs are becoming the first-choice treatment for immature teeth diagnosed with pulp necrosis and apical periodontitis^29^. In addition, infected immature teeth with endodontic disorders often exhibit some remnant pulp and apical tissue^10–14^. Therefore, this study investigated the impacts of remnant healthy or fully functional pulp and apical tissue on healing patterns after REPs. In this study, simulated REPs were performed on non-infected immature rat molars with different amounts of remnant healthy pulp and apical tissue. The systemic effects of treatment are likely to be negligible during healing after REPs because the contralateral untreated control teeth developed in a standard physiological manner (Fig. 1I,J). These REP-treated teeth exhibited new mineralized tissue formation within 1 month after REPs, although previous REP experiments using larger animals have required healing periods of > 3 months to observe new mineralized tissue formation after REPs^15–17^. The more rapid progression of healing is an advantage of using a rat model. Histological and immunohistochemical analyses of these REP-treated teeth revealed that four distinct healing patterns occurred after REPs, depending on the preoperative amount of remnant healthy pulp and apical tissue.

In past investigations, mineralized tissues formed after REPs were reported to include dentin, DAMT, and IB^15,27^; notably, DAMT and IB are sometimes given different names. In the present study, two subtypes of DAMT were distinguished: DSP-positive OSD-DAMT and DSP-negative CEM-DAMT. DSP expression in DAMT or DAMT-equivalent tissue was analyzed in three previous studies; however, the subtypes of DAMT have not been previously identified. Yamauchi et al. reported that DAMT was immunopositive for DSP^27^, while Austah et al. and Zhu et al. reported that DAMT-equivalent tissue was immunonegative for DSP^2,28^. Presumably, only OSD-DAMT was formed in the study by Yamauchi et al., while only CEM-DAMT was formed in the studies by Austah et al. and Zhu et al.

The results of the present study suggest that pulp-like tissue reconstruction after REPs is associated with the amount of remnant pulp tissue; it may occur if more than a specific amount of remnant healthy pulp (i.e., above an unknown threshold) remains preoperatively, in contrast to the replacement by PDL-like tissue that may develop if the preoperative amount of remnant healthy pulp is below the threshold. Thus, it may be reasonable to hypothesize that remnant healthy pulp plays a role in preventing intracanal invasion by PDL cells. However, more research is needed to test this hypothesis.

When pulp-like tissue was reconstructed after REPs, formation of dentin and OSD-DAMT or formation of OSD-DAMT alone occurred. Considering that the histological and immunohistological features of OSD-DAMT were identical to those of osteodentin (Fig. 1T,U and Fig. 2Be,Bf,Ce–Ch,Dg,Dh), the cause of OSD-DAMT formation may be identical to that of osteodentin formation. Osteodentin is known to be formed by pulp cell-derived cells if normal odontoblast differentiation is disturbed by inadequate signals from the surrounding environment^30,31^. Therefore, the microenvironment in reconstructed pulp-like tissue may determine the fate of the pulp cells, as well as whether dentin or OSD-DAMT is formed in the tissue. We found that the preoperative amount of remnant healthy pulp was greater in teeth with newly formed dentin and OSD-DAMT than in teeth with OSD-DAMT alone (Table 1). Presumably, it is more difficult for a smaller amount of remnant healthy pulp to provide a suitable microenvironment for odontoblast differentiation during healing after REPs. In this study, ideal dentin-pulp regeneration without any ectopic tissue formation did not occur after simulated REPs, even in teeth with some amount of remnant healthy pulp. Conversely, Torabinejad et al. reported that ideal dentin-pulp regeneration was achieved after simulated REPs in immature ferret teeth if a small amount of remnant healthy pulp was present in the teeth preoperatively^32^. This discrepancy is presumably because a suitable microenvironment for odontoblast differentiation could not readily be established by using our protocol for simulated REPs, compared with the protocol described by Torabinejad et al. In the present study, root canals were irrigated with sodium hypochlorite, whereas no irrigation was used in the prior study. Sodium hypochlorite treatment of dentin has been shown to negatively influence odontoblast differentiation on the dentin surface^33,34^. Furthermore, in the present study, an endodontic file was inserted beyond the root apex to produce an intracanal blood clot, whereas no overinstrumentation was performed in the prior study. Overinstrumentation may damage remnant pulp tissue and disturb odontoblast differentiation.

When PDL-like tissue replaced intracanal tissue after simulated REPs, formation of CEM-DAMT and OSD-DAMT or formation of CEM-DAMT and IB occurred. CEM-DAMT was formed only when PDL-like tissue replaced intracanal tissue (Table 1). In addition, the histological and immunohistological features of CEM-DAMT were identical to those of cementum (Fig. 1E,M and Fig. 2De,Df,Ee,Ef). Therefore, CEM-DAMT may be formed by cementoblast-like cells that differentiate from intracanal-invading PDL cells. Furthermore, we found that teeth with a marginal amount of remnant healthy pulp exhibited formation of CEM-DAMT and OSD-DAMT, while teeth with disorganization of pulp and apical tissues beyond the root apex exhibited formation of CEM-DAMT and IB (Table 1). These findings indicate that remnant pulp is necessary for OSD-DAMT formation, supporting our hypothesis that OSD-DAMT is formed by pulp cell-derived cells. Additionally, these findings imply that loss of whole pulp tissue and/or loss of a specific type of apical tissue before REPs may trigger IB formation. In the present study, loss of HERS was observed with IB formation (Fig. 2Eh). A previous study showed that alveolar bone grew into the root canal space of auto-transplanted immature monkey teeth if HERS was removed from the teeth before transplantation^35^. Furthermore, epithelial rests of Malassez, which comprise fragments of HERS, have been proposed to suppress bone formation within PDL tissue by releasing matrix metalloproteinases^36,37^. Therefore, the loss of HERS may allow IB formation in intracanal PDL-like tissue of REP-treated teeth.

This study evaluated how the healing pattern after REPs was influenced by the preoperative amount of remnant healthy pulp and apical tissue. However, the distribution of stem cells with odontoblastic differentiation potential (dental pulp stem cells and stem cells from apical papilla) is not homogenous within pulp and apical tissue. Notably, stem cells from apical papilla have greater proliferation and mineralization abilities than dental pulp stem cells; moreover, stem cells from apical papilla are only located around the root apex^38^. Therefore, the relationship between the outcome after REPs and the preoperative amount of remaining stem cells with odontoblastic differentiation potential should be investigated in future studies.

Collectively, this study revealed that four distinct healing patterns occurred after REPs in immature rat molars, depending on the preoperative amount of remnant healthy pulp and apical tissue. Pioneering studies of REPs, published from 1961 to 1971, demonstrated that bleeding from the root apex induces neo-tissue formation in the pulpotomized root canal space^39–41^. Moreover, there has been considerable effort over the past two decades to clarify the healing mechanisms after REPs. However, these healing mechanisms have not been fully elucidated. The findings in this study provide new insights regarding the mechanisms that influence healing patterns after REPs. Our findings suggest that measurement of the preoperative amount of remnant pulp and apical tissue may be useful for clinical outcome prediction after REPs. Moreover, our findings suggest that minimizing injury to remnant pulp and apical tissue during REPs might be effective for inducing better healing patterns after REPs; specifically, dentin formation is ideal, DAMT formation is acceptable, and IB formation is undesirable. However, caution is needed regarding interpretation of the present results, because the simulated REPs performed in this study were distinct from actual REPs in terms of the absence of infection. Additional studies are needed to fully elucidate the mechanisms that influence healing patterns when REPs are performed on immature teeth diagnosed with pulp necrosis and apical periodontitis. Notably, the degree of preoperative inflammation^42^, type of disinfectant agents used before REPs^43^, and presence or absence of postoperative residual bacteria^12^ also seem to influence the healing pattern after REPs. Our findings may serve as the foundation for future studies.

## Methods

### Animals and treatments

All experiments were approved by the Committee on the Guidelines for Animal Experimentation of Niigata University, Niigata, Japan, and performed in accordance with the recommendations of the Committee (approval no. SA00213). Twenty-six 5-week-old male Wistar rats were purchased from Clea Japan (Tokyo, Japan). These rats are known to exhibit immature mandibular first molars at 5 weeks of age^18^. Two rats were sacrificed immediately to obtain baseline samples of mandibular first molars. Four rats underwent pulpotomy as described in the supplementary information and were sacrificed after the 28-day experiment to obtain samples for characterization of mineralized tissues formed during pulp wound healing. Twenty rats underwent simulated REPs.

Protocols for simulated REPs were as follows. General anesthesia was induced with sevoflurane and chloral hydrate. Surgical procedures were performed under 20× magnification (S9D microscope; Leica, Wetzlar, Germany). Under rubber dam isolation with a custom-made rubber dam clamp (YDM, Tokyo, Japan) and a rubber dam sheet, the left mandibular first molars were accessed through the occlusal surface into the pulp chamber using a #1/2 round carbide bur attached to a contra-angle handpiece. Subsequently, pulp and apical tissues of the mesial roots were removed at four different levels (n = 5 rats per level) by instrumentation using a #25 K-file and syringe irrigation with 1 ml of room temperature 2.5% sodium hypochlorite. The levels of tissue removal were set such that approximately 0.8 mm, 0.5 mm, or 0.2 mm of tissue remained inside the root canal, or such that 0.2 mm of tissue was removed beyond the root apex, based on the average root length determined in a previous study^18^. The prepared root canals were then filled with X-ray contrast media (Visipaque; Daiichi Sankyo, Tokyo, Japan), and scanned by micro-CT (CosmoScan GX; Rigaku, Tokyo, Japan). The X-ray contrast media was carried into the root canal by using endodontic irrigation syringe and a needle; during the micro-CT scans, the X-ray energy was set at 90 kV and 88 µA, with an exposure time of 2 min. Pre-REP micro-CT images were used to measure the amount of remnant pulp and apical tissue, as described later in the Methods section. After micro-CT scans had been performed, root canals were irrigated with 5 ml of room temperature sterile saline and dried using sterile paper points; intracanal blood clot formation was then induced by overinstrumentation with a #10 H-file. Thereafter, a calcium-silicate cement (ProRoot MTA; Dentsply Sirona, York, PA, USA) was gently placed on the blood clot by using an MTA applicator (Japan Dental Supply; Tokyo, Japan) with a thin root canal plugger (YDM); the cavity was immediately sealed with a bonding system (Clearfil Universal Bond Quick; Kuraray, Tokyo, Japan) and a flowable composite resin (MI Fill; GC, Tokyo, Japan). Right mandibular first molars were used as untreated controls. Observations were made at 28 days after treatment.

### Tissue preparation

Rats were transcardially perfused with 4% paraformaldehyde under general anesthesia. Subsequently, mandibular first molars were removed with surrounding tissue and immersed in 4% paraformaldehyde for an additional 24 h. Micro-CT scans were again performed with the X-ray energy set at 90 kV and 88 µA, with an exposure time of 14 min; samples were decalcified with 10% ethylenediaminetetraacetic acid solution and embedded in paraffin blocks. The paraffin-embedded samples were sectioned at a thickness of 5 µm using a microtome.

### Histological and immunohistochemical staining

For hematoxylin–eosin staining, Mayer's hematoxylin (Wako, Tokyo, Japan) and 1% eosin Y (Wako) were used. For TRAP staining, a TRAP staining kit (Wako) was used in accordance with the manufacturer’s protocol. Immunohistochemical staining was performed as described in our previous study^44^. Primary antibodies used in the present study were a mouse anti-DSP monoclonal antibody (clone 2C12.3; Sigma-Aldrich, St. Louis, MO, USA), a mouse anti-nestin monoclonal antibody (clone rat-401; Sigma-Aldrich), a rabbit anti-periostin polyclonal antibody (BioVendor, Karasek, Czech Republic), and a rabbit anti-cytokeratin polyclonal antibody (Nichirei Bioscience, Tokyo, Japan). Negative control staining was performed by replacing primary antibodies with non-immune mouse or rabbit IgG (Santa Cruz Biotechnology, Dallas, TX, USA). The control sections did not show any specific immunoreactivity.

### Measurement of preoperative amount of remnant pulp and apical tissue

Pre-REP micro-CT images were standardized as shown in Fig. 3, using the Three-Dimensional Reconstruction Imaging for Bone system (Ratoc System Engineering, Tokyo, Japan). In the images, the distance from the apical foramen to the bottom of the contrast-enhanced area was regarded as the amount of remnant pulp and apical tissue. A sample number was given to each REP-treated tooth according to the amount of remnant pulp and apical tissue, in a descending manner.

## Supplementary information

Supplementary Information.

## Data Availability

The datasets generated during and/or analyzed during the current study are available from the corresponding author on request.
