# Supplementary material for: Impact of remnant healthy pulp and apical tissue on outcomes after simulated regenerative endodontic procedure in rat molars
Source: Sci Rep. 2020 Dec 1;10:20967. doi: 10.1038/s41598-020-78022-w (PMC7708843; doi:10.1038/s41598-020-78022-w)
Supplement: Supplementary file 1 — Supplementary Information. [file 41598_2020_78022_MOESM1_ESM.pdf]

Supplementary information

Impact of remnant healthy pulp and apical tissue on outcomes after simulated regenerative endodontic procedure in rat molars

Naoki Edanami[1]*, Kunihiko Yoshiba[2], Mari Shirakashi[1], Razi Saifullah Ibn Belal[1], Nagako Yoshiba[1], Naoto Ohkura[1], Aiko Tohma[1], Ryosuke Takeuchi[1], Takashi Okiji[3] and Yuichiro Noiri[1].

[1]Division of Cariology, Operative Dentistry and Endodontics, Department of Oral Health Science, Niigata University Graduate School of Medical and Dental Sciences, Niigata, Japan

[2]Division of Oral Science for Health Promotion, Department of Oral Health and Welfare, Niigata University Graduate School of Medical and Dental Sciences, Niigata, Japan

[3]Department of Pulp Biology and Endodontics, Division of Oral health Sciences, Graduate School of Medical and Dental Sciences, Tokyo Medical and Dental University (TMDU), Tokyo, Japan.

*: Corresponding author:
Naoki Edanami,
Division of Cariology, Operative Dentistry and Endodontics
Department of Oral Health Science, Course for Oral Life Science
Niigata University Graduate School of Medical and Dental Sciences
2-5274 Gakkocho-dori, Chuo-ku, Niigata 951-8514, Japan
Phone: +81-25-227-2866; Fax: +81-25-227-2864; E-mail: edanami@dent.niigata-u.ac.jp

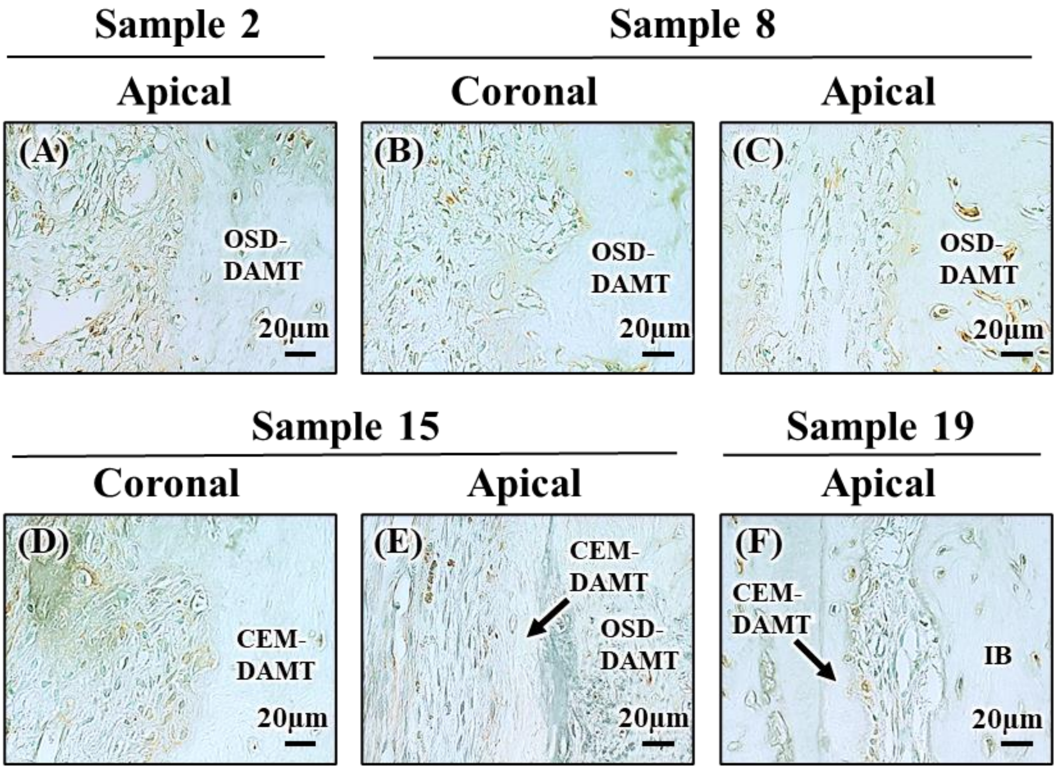

Supplementary figure S1

Nestin immunostaining of regenerative endodontic procedure-treated teeth. Sample numbers and regions are indicated. No nestin-positive odontoblasts are observed on the surfaces of osteodentin-like dentin-associated mineralized tissue (OSD-DAMT) (A–C), cementum-like dentin-associated mineralized tissue (CEM-DAMT) (D–F), or intracanal bone (IB) (F).

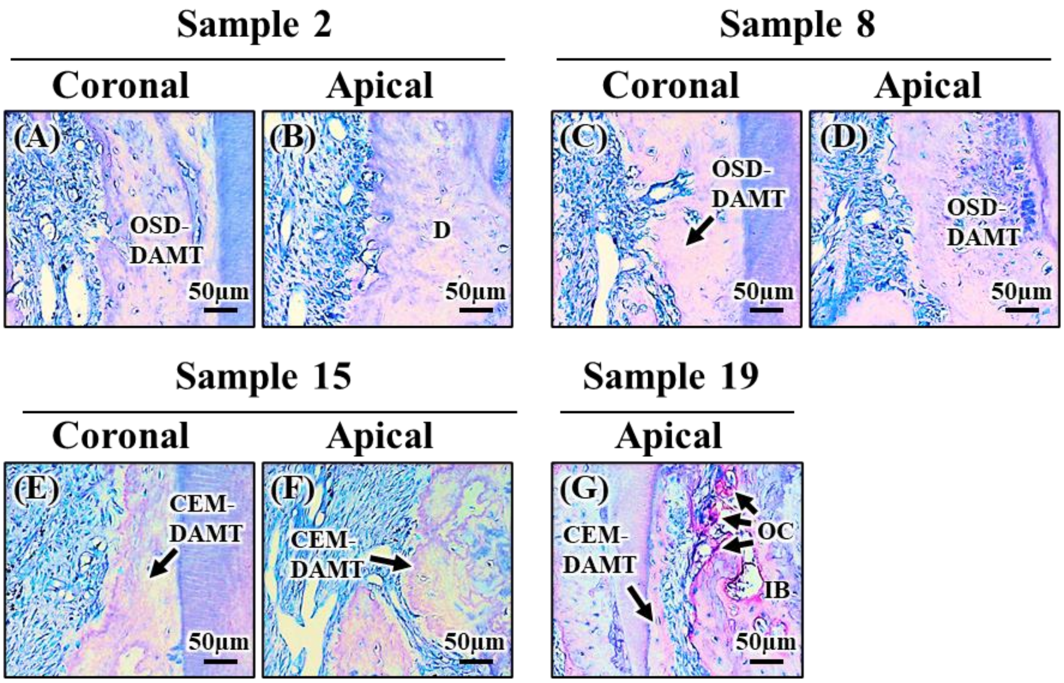

Supplementary figure S2

Tartrate-resistant acid phosphatase (TRAP) staining of regenerative endodontic procedure-treated teeth. Sample numbers and regions are indicated. No TRAP-positive osteoclasts are observed on the surfaces of newly formed dentin (B), osteodentin-like dentin-associated mineralized tissue (OSD-DAMT) (A, C, D), or cementum-like dentin-associated mineralized tissue (CEM-DAMT) (E–G). D: dentin, IB: intracanal bone, OC: osteoclast.

Methods

Pulpotomy procedures.

Pulpotomy procedures were performed as written in our prior study[1]. Under general anesthesia with sevoflurane and chloral hydrate, the dental pulp of the lower first molar was exposed at the occlusal surface; coronal pulp tissue was removed using a #1/2 round carbide bur. The exposed area was rinsed with 2.5% sodium hypochlorite, followed by sterile saline. Hemorrhaging was controlled with sterile cotton pellets. A calcium-silicate cement (ProRoot MTA; Dentsply Sirona, York, PA, USA) was then mixed and placed over the pulp stump. The cavity was sealed with a bonding system (Clearfil Universal Bond Quick; Kuraray, Tokyo, Japan) and a flowable composite resin (MI Fill; GC, Tokyo, Japan).

References

1. Edanami, N. et al. Characterization of dental pulp myofibroblasts in rat molars after pulpotomy. *J. Endod.* **43**, 1116–1121 (2017).
